# Forensic age assessment of the knee: proposal of a new classification system using two-dimensional ultrasound volumes and comparison to MRI

**DOI:** 10.1007/s00330-020-07343-1

**Published:** 2020-10-14

**Authors:** Jochen Herrmann, Dennis Säring, Markus Auf der Mauer, Michael Groth, Eilin Jopp-van Well

**Affiliations:** 1 Department of Diagnostic and Interventional Radiology and Nuclear Medicine, Section of Pediatric Radiology, University Medical Center Hamburg-Eppendorf (UKE), Martinistrasse 52, 20246 Hamburg, Germany; 2 Department of Medical and Industrial Image Processing, University of Applied Sciences of Wedel, Feldstraße 143, 22880 Wedel, Germany; 3 Department of Legal Medicine, University Medical Center Hamburg-Eppendorf (UKE), Butenfeld 34, 22529 Hamburg, Germany

**Keywords:** Adolescent, Growth plate, Ultrasonography, Magnetic resonance imaging, Knee joint

## Abstract

**Objectives:**

To assess epiphyseal growth plate closure of the knee for forensic age estimation using an ultrasound (US)-based method and to compare the findings with MRI.

**Methods:**

Thirty-three healthy male individuals (age, 14.4–19.3 years) were prospectively evaluated for epiphyseal growth plate closure of the right knee by recordings of two-dimensional US volumes and a high-resolution T1-weighted MRI sequence. The degree of epiphyseal growth plate closure was rated independently by two readers for each method using a modality specific three-point scale that differentiates between an open physis (S1), a partially closed physis (S2), and a closed physis (S3).

**Results:**

The inter-rater agreement was high for the US (Cohen’s kappa (CK): femur 95.2%, tibia 81.3%, fibula 86.3%) and the MRI method (CK: femur 70.2%, tibia 90.8%, fibula 79.8%). The degree of growth plate closure associated positively with advancing age. The US system showed a clearer separation of median ages with lower overlap than the MRI system. Open growth plates on minors (< S3 on femur and tibia) were identified by US with higher sensitivity (1.0 vs. 0.7) and slightly lower specificity (0.7 vs. 0.85) compared with MRI. The examination time was substantially shorter on US than on MRI (2.65 ± 0.91 min vs. 24.72 ± 2.72 min; *p* < 0.001).

**Conclusions:**

The US method for evaluation of growth plate closure of the knee can reliably assign male individuals to different ossification stages and identifies minors with high accuracy. More studies with larger numbers are needed to further evaluate this method.

**Key Points:**

*• US is feasible to determine the degree of epiphyseal growth plate closure of the knee, shows a high degree of reliability, and is comparable to MRI.*

*• US of the knee can detect open growth plates on male minors with high accuracy.*

*• US of the knee may be used as a fast, non-invasive imaging tool for forensic age estimation to identify male minors*.

**Electronic supplementary material:**

The online version of this article (10.1007/s00330-020-07343-1) contains supplementary material, which is available to authorized users.

## Introduction

Age estimation in forensic medicine has gained increasing importance since the 1990s due to high levels of immigration into Europe and is an element of foreigner, asylum, and criminal law [1]. Depending on the country, the age for criminal liability ranges from 14–21 years of age [2, 3]. The European asylum procedure states that individuals < 18 years of age are minors, thus different rights and benefits apply to minors than to adults [3, 4].

Age assessment has to be safeguarded in the best interest of the potential child. If the age of an individual cannot be determined by non-medical methods alone, medical imaging may be used in accordance with the local legal provisions beginning with an x-ray of the left hand or a dental view. When ossification is completed, a computer tomography (CT) of the clavicles is recommended to specify an age > 18 years [5]. However, the application of ionizing radiation for non-medical purposes has been criticized due to its potentially harmful effects [4] and due to the availability of alternative radiation-free methods.

Magnetic resonance imaging (MRI) has been evaluated for age estimation in a variety of anatomical regions [6–40]. The knee has three neighboring growth plates and the extent of epiphyseal closure carries important information about the person’s age [41]. On x-ray, a complete bony fusion is seen in the knee beginning with 14 years in females and 15–16 years in males. Epiphyseal closure is recognized later on MRI and explained by better delineation of small growth plate remnants [34, 36]. However, it has not been consistently shown that a complete bony fusion on knee MRI can be used to define majority (age > 18) due to overlap with younger age groups [39]. Also, the applicability of MRI for forensic age estimation has been questioned because of high costs, relatively long duration, and the potential stress the examination may induce in traumatized children [42].

Recently, ultrasound (US) has been shown to be a cost-effective, alternative method for forensic age determination avoiding ionizing radiation [43–47]. The US examination is well tolerated and the preferred imaging modality in child medicine. Therefore, the method seems to be especially suited for examination of unaccompanied minors. In pilot studies, US has been used for age determination inspecting the iliac crest [48, 49], the elbow [50], the hand [51–55], and the clavicle [56–58], as well as the femoral trochanter [59]. Reported limitations of the US method included accessibility and overlay resulting in reduced reliability [4]. Depending on the area under inspection, increased ossification with age hindered the imaging of deeper bony structures and led to incomplete documentation of regions, such as the clavicular epiphysis [4]. To our knowledge, US has not been used for forensic age estimation of the knee. Based on systematic studies in healthy children and clinical experiences in traumatology and rheumatology, it is known that the bony and soft tissue structures of the knee are clearly visible and well defined in US images [60, 61].

The aim of this study was to test the feasibility of an US-based method for assessment of epiphyseal growth plate closure around the knee for forensic age estimation and to compare the findings to MRI.

## Material and methods

### Study population

This prospective study was approved by the institutional review board and written informed consent was obtained from all of the participants. To methodically test MRI and US of the knee for age determination, a homogenous study population was defined and limited to healthy, male individuals between 14–19 years of age. At our institution, forensic age assessment is mainly requested for male subjects. All participants of the study lived near Hamburg, Germany, had a middle–high socioeconomic status, and were recruited from staff. A total of 40 male individuals (mean age 16.7 ± 1.6 years) were examined between April–June 2015 for epiphyseal closure of the right knee. Exclusion criteria included the presence of fractures around the right knee, a chronic disease that potentially affected bone growth, and incomplete documentation. Of the 40 scheduled cases, 4 MRI examinations were canceled on the day of the examination due to non-MRI-compatible implants. In 4 cases, documentation of the US examination was incomplete. In total, 33 cases with full MRI and US data sets were included in this study (mean age 15.5 years, range 14.4–19.3 years; Table 1).

**Table 1:** Characteristics of the included subjects

Data	Min	Max	Mean ± SD
Age (years)	14.4	19.3	16.5 ± 1.5
Weight (kg)	49.2	117.0	68.4 ± 13.9
Height (cm)	154.5	196.0	177.2 ± 7.8

The chronological age for each subject and time point was calculated as the difference between the date of birth and the imaging acquisition date. Following the conservative estimate used for forensic age assessment, the chronological age was rounded to full years, for example, 17.8 years was rounded to 17 years. The data sets were made anonymous in a preprocessing step. The time required to perform each method was based on the duration on the examination table (the difference between the times of the first and last recorded images) and the time to perform an US and an MRI were compared.

### US protocol

**Fig. 1:**
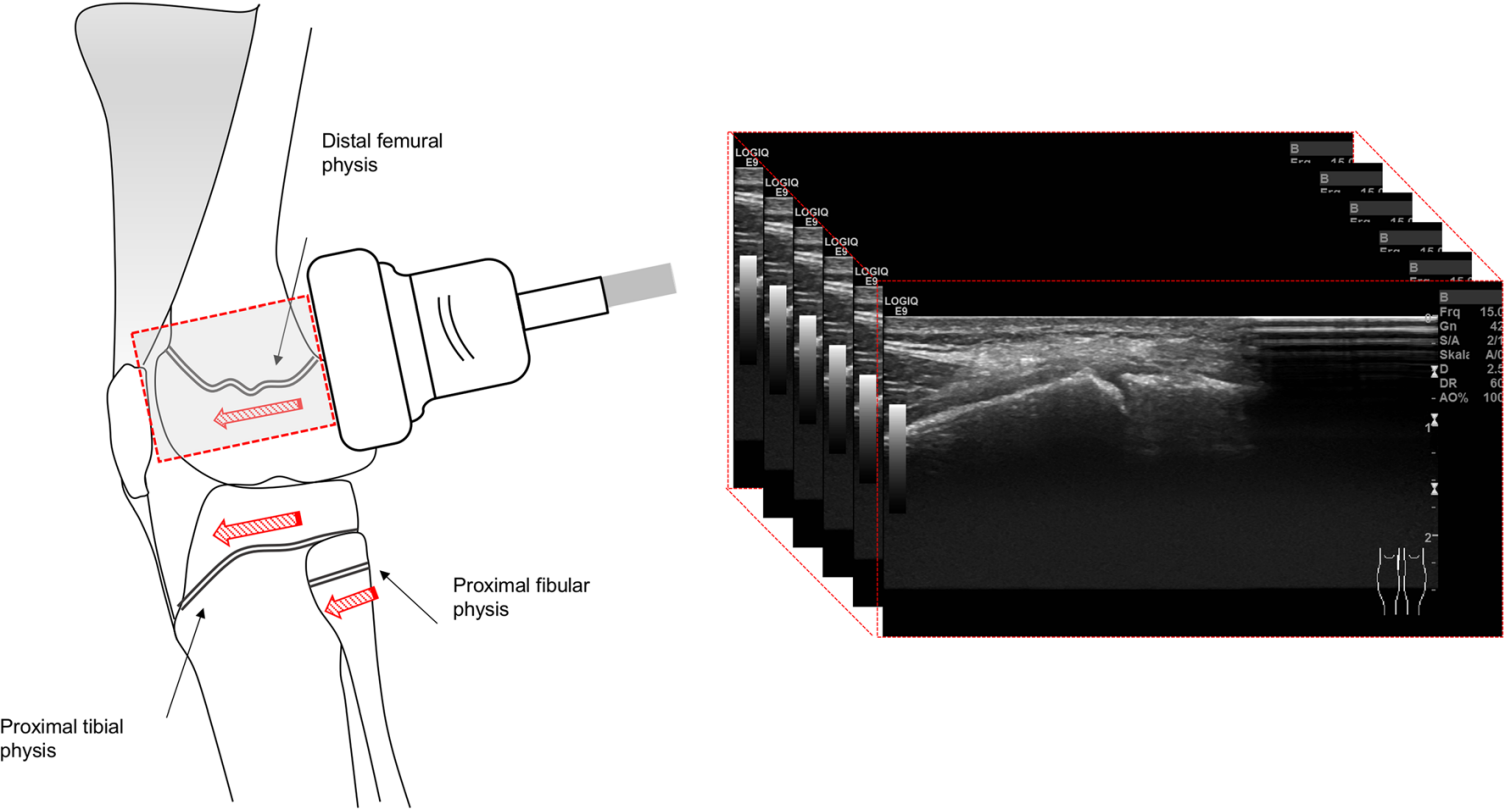
Illustration of the US examination of the knee. To record 2D-US volumes, the linear probe was placed longitudinally and moved slowly from the dorsal to the frontal growth plate end. The distal femoral and the proximal tibial physis were examined from the medial and lateral side. The fibular physis only from the lateral position

US examinations were performed on the same day as the MRI by one of two radiologists (J.H., M.G.) on a commercially available scanner (Logiq E9, GE Healthcare) using a high-resolution linear transducer (ML6-15D, 4–15 MHz, 50 mm). The distal femoral and the proximal tibial physis were assessed with medial and lateral positioning of the probe. Due to the anatomy, the proximal fibular physis was only assessed from a lateral position. Representative B-mode images were acquired from the central part of the physis. Additionally, longitudinally oriented B-mode two-dimensional (2D) volumes in the same plane were recorded from the dorsal part to the ventral part of each physis (Fig. 1). In total, five representative single images and five 2D volumes were documented for each case (medial distal femoral physis, lateral distal femoral physis, medial proximal tibial physis, lateral proximal tibial physis, and lateral proximal fibular physis).

### MRI protocol

MRI was performed on a 3 T-MRI scanner (Ingenia 3.0, Philips Healthcare) using a knee coil (8-Channel-Knee-Coil, Philips Healthcare). The protocol included a 3D-T1-weighted FFE sequence in transverse orientation (TR 6.1 ms, TE 2.3 ms, flip angle 35°, field of view 180 × 180, in-plane resolution 0.625 × 0.625 mm^2^, slice thickness 1.2 mm, spacing between slices 0.6 mm), a T1-weigthed TSE in sagittal (TR 1120 ms, TE 10.8 ms, flip angle 90°, FOV 150 × 150, in-plane resolution 0.174 × 0.174 mm^2^, slice thickness 2.0 mm, spacing between slices 2.2 mm), and coronal orientation (TR 855 ms, TE 10.8 ms, flip angle 90°, FOV 150 × 150, in-plane resolution 0.188 × 0.188 mm^2^, slice thickness 2.0 mm, spacing between slices 2.2 mm).

### Grading of growth plate closure

#### US

**Fig. 2:**
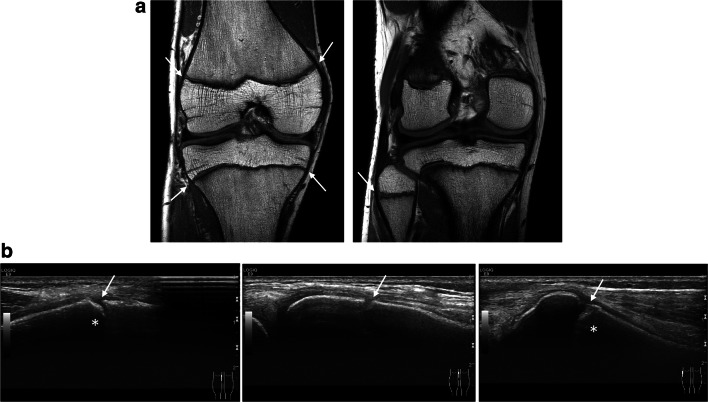
Right knee of a 15-year-old boy showing wide open physeal plates (arrows) on a coronal T1-weighted MRI (**a**) and longitudinally oriented US images (**b**) on the medial distal femur (left image), the medial proximal tibia (middle image), and the proximal fibula (right image). On the US, note the gap between the metaphyseal and epiphyseal cortex and the linear, echogenic zone of calcification continuing transversally into the physis (asterisk, US-S1)

**Fig. 3:**
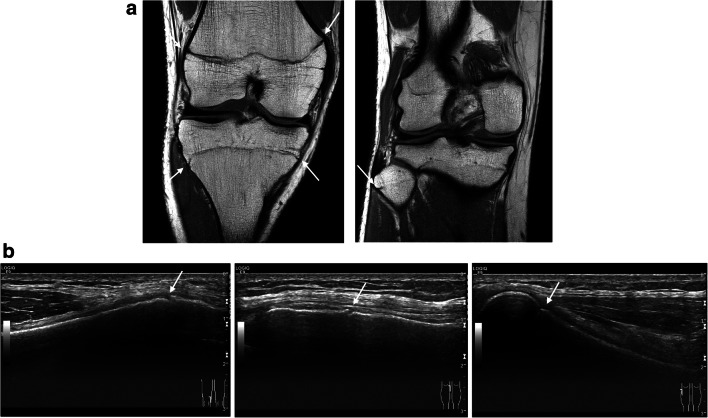
Right knee of a 17-year-old boy showing slightly open physeal plates (arrows) on a coronal T1-weighted MRI (**a**) and longitudinally oriented US images (**b**) on the medial distal femur (left image), the medial proximal tibia (middle image), and the proximal fibula (right image). On the US, note only the small gap between the metaphyseal and epiphyseal cortex, the physeal plate is not visible (US-S2)

**Fig. 4:**
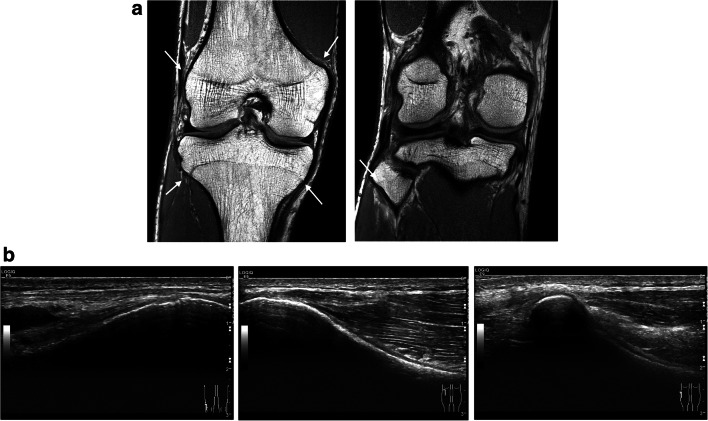
Right knee of a 17-year-old boy showing closed physeal plates (arrows) on a coronal T1-weighted MRI (**a**) and longitudinally oriented US images of the lateral distal femur (right image), the lateral proximal tibia (middle image), and the proximal fibula (left image). On the US, note the closed echogenic line of the cortical bone (US-S3)

For US-based visual grading of growth plate closure around the knee, a three-point scale was developed with defined criteria (Table 2). Exemplary cases illustrating US and MRI stage 1–3 definitions are shown in Figs. 2, 3, and 4 (electronic supplementary material, 2D-US volumes representing US-S1–3 cases shown in Figs. 2, 3, and 4). Grading was performed offline on a PACS station by two raters (J.H., M.G.) independently. Both readers were fully blinded regarding information on the subjects and the results of the MRI examinations.

**Table 2:** US-based staging of growth plate closure around the knee

US-based visual staging of growth plate closure of the knee joint	
US-S1	Open growth plate with a large gap between the metaphysis and the epiphysis (2–3 mm) visible on all images, right angle step-off from the outer cortex into the physis representing the metaphyseal provisional zone of calcification
US-S2	Small diameter growth plate with only a small, shallow notch between the metaphysis and the epiphysis, which may not be seen on all 2D volume images (> 50% of images)
US-S3	Closed growth plate with no gap between the epiphysis and the metaphysis

Note: Staging was performed on standardized longitudinally oriented 2D volumes. In cases of discrepancies between medial and lateral volumes of femoral and tibial growth plates, the lower category was used

#### MRI

MRI-based grading of the physeal closure was performed by two independent readers (E.J., M.M., each with 5 years of experience in forensic medicine) according to the method published by Jopp et al [35, 62] using a three-stage visual grading system (Table 3). As described for the US-based approach, the degree of physeal closure was graded for the distal femur, the proximal tibia, and the proximal fibula. The grading was performed on visually preselected coronal images representing the center of the bone. For both the femur and the tibia, one slice was used. The fibula was rated based on a different slice to capture the center of the bone. The selection was also performed by visual inspection.

**Table 3:** MRI-based staging system of growth plate closure around the knee according to Jopp et al [35]

MRI-based visual staging of growth plate closure of the knee joint	
MRI-S1	Open growth plate and a broad, T1w hypointense band is noted between the epiphysis and the metaphysis
MRI-S2	Partially fused growth plate in the center of the bone, the remnant of the physeal plate can be identified by a very thin line representing the epiphysical scar, and the peripheral growth plate is not fused
MRI-S3	Closed growth plate, the physis is completely fused from the center to the periphery, and only traces of an epiphysical scar may be visible

Note: Staging was performed on coronally oriented T1-weighted images of the center of the bone

### Statistical analysis

Statistical analyses of the inter-rater agreement, the correlation between MRI and US staging, and the correlation between staging and age estimation were performed using R software (v 3.0.2) [63]. Inter-rater agreement was analyzed using calculated values for the percentage of agreement (PoA) and Cohen’s kappa (CK). Correlation analysis was performed two-sided and evaluated at the 5% significance level using the Pearson correlation coefficient. The durations of the US and MRI examinations were compared using the two-sided *T* test.

## Results

Standardized US volumes and corresponding MRI planes of the right knees of 33 male adolescents and young adults were analyzed. Per subject, five high–spatial resolution US volumes and one high-resolution MRI T1-sequence were assessed independently by two raters. The image quality of all the examinations was good and no abnormalities were noted that required exclusion from the study.

### US

US volumes of the distal femur and the proximal tibia were recorded from medial and lateral probe positions. No differences in maturation were found between the medial and lateral views for each anatomical region per individual case. The agreement between readers for all three anatomical regions was high (CK: distal femur 95.2%, proximal tibia 81.3%, proximal fibula 86.3%). The best PoA was noted for US-S1 (completely open physis) and US-S3 (completely closed physis) cases (Table 4). The assignment of US-S2 (partly open physis) for the tibia and fibula had lower inter-reader PoA values.

**Table 4:** Inter-rater agreement for the US- and MRI-based staging systems

	Femur (%)	Tibia (%)	Fibula (%)
PoA for all US stages	97.1	88.6	91.4
US-S1	100	100	94.6
US-S2	95.7	55.6	88.9
US-S3	100	100	100
CK	95.2	81.3	86.3
PoA for all MRI stages	80.0	91.4	85.7
MRI-S1	94.4	92.9	100
MRI-S2	50	90.9	66.7
MRI-S3	85.7	100	93.3
CK	70.2	90.8	79.8

Note: *CK* Cohen’s kappa = 0% (no agreement), 0–20% (poor agreement), 20–40% (fair agreement), 40–60% (moderate agreement), 60–80% (good agreement), and > 80% (very good agreement). *US* ultrasound; *US-S1* US stage 1, open physis; *US-S2* US stage 2, partly closed physis; *US-S3* US stage 3, closed physis. *MRI* magnetic resonance imaging; *MRI-S1* MRI stage 1, open physis; *MRI-S2* MRI stage 2, partly closed physis; *MRI-S3* MRI stage 3, closed physis; *PoA* best percentage of agreement

**Fig. 5:**
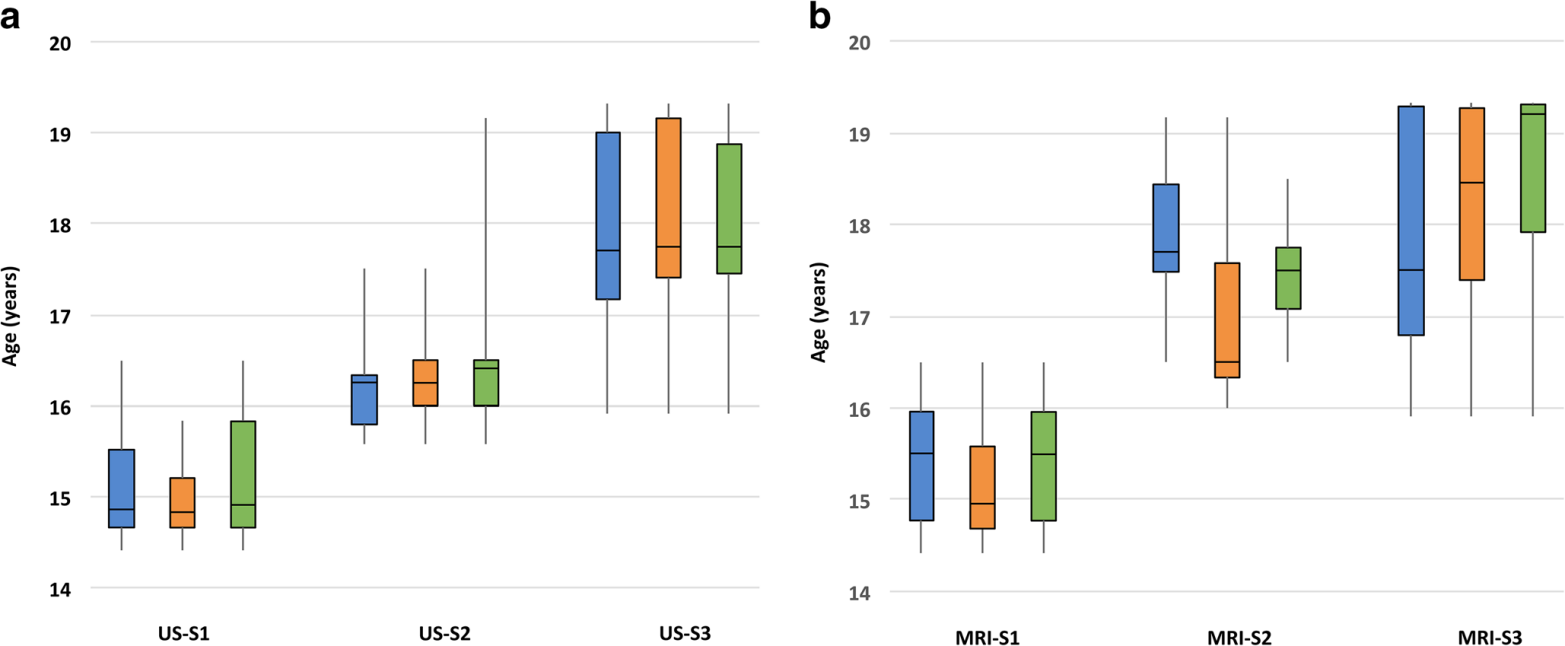
**a** Box-plot of the US-based stage definitions (US-S1–3) according to chronological age (femur, blue; tibia, orange, fibula, green). **b** Box-plot of the MRI-based stage definitions (MRI-S1–3) according to chronological age (femur, blue; tibia, orange, fibula, green)

The degree of growth plate closure determined by US associated positively with advancing age (Pearson correlation coefficient: distal femur 0.82, proximal tibia 0.83, proximal fibula 0.80). Median ages of subjects with US-S1, US-S2, and US-S3 (femur, tibia) were 14.9 years (range, 14.4–16.5 years), 16.3 years (range, 15.6–17.5 years), and 17.7 years (range, 15.9–19.3 years), respectively (Fig. 5).

### MRI

Using the MRI staging system, the inter-rater agreement for all three anatomical regions was also high (CK: distal femur 70.2%, proximal tibia 90.8%, proximal fibula 79.8%). Comparable to the US results, the agreement between readers was better for stage 3 and stage 1 (completely closed physis and completely open physis; Table 2). The assignment of stage 2 (partly open physis) showed lower PoA values.

A higher stage associated positively with advancing age (Pearson correlation: distal femur 0.78, proximal tibia 0.80, proximal fibula 0.84). The median age of subjects with MRI-S1, MRI-S2, and MRI-S3 was 15.5 years (range, 14.4–16.5 years), 17.7 years (range, 16.5–19.2 years), and 18.0 years (range, 15.9–19.3 years), respectively (Fig. 5b).

### US vs. MRI

The correlation between MRI and US staging was very good (Pearson correlation coefficient: distal femur 0.84, proximal tibia 0.87, proximal fibula 0.80). All cases with an open physis on MRI (MRI-S1) were also rated as open by US, for example, for the femur, 12/12 cases agreed 100% (Table 5). The largest disagreement between the methods was for cases with stage 2 classification (partially closed physis). US slightly outperformed MRI with a higher sensitivity (sens_US_ = 1.0 vs. sens_MRI_ = 0.7), but slightly lower specificity (spec_US_ = 0.7 vs. spec_MRI_ = 0.85) and accuracy (acc_US_ = 0.76 vs. acc_MRI_ = 0.85) in the identification of minors (age < 18) using stage 2 threshold (tibia and femur) data. For the ability of the US and MRI methods to identify age groups based on visual evaluation, US staging showed a clearer separation of ages with lower overlap (Fig. 5 a and b). The US examination was also 9.3 times shorter than MRI (2.65 ± 0.91 min vs. 24.72 ± 2.72 min, *p* < 0.001, *t* = 24.4, CK of 99%).

**Table 5:** Agreement of US- to MRI-based stages in the three anatomical regions

	S1—open physis			S2—partially closed physis			S3—closed physis		
	Femur	Tibia	Fibula	Femur	Tibia	Fibula	Femur	Tibia	Fibula
Correct	12	11	13	1	6	3	7	8	5
US-S < MRI-S	0	0	0	0	0	1	0	0	0
US-S > MRI-S	0	0	0	6	3	5	7	5	6
Total (*n*)	12	11	13	7	9	9	14	13	11
Total false	0	0	0	6	3	6	7	5	6
PoA	100%	100%	100%	14.3%	66.7%	33.3%	50.0%	61.5%	45.5%

Note: *US-S* ultrasound stage; *MRI-S* MRI stage; *US-S < MRI-S* US stage lower than the MRI stage; *US-S > MRI-S* US stage greater than the MRI stage; *PoA* best percentage of agreement

## Discussion

This work investigated the feasibility of an US-based method for forensic age estimation and compared the findings with MRI. To our knowledge, this is the first study to evaluate US of the knee as an alternative to other cross-sectional imaging methods for age determination.

Previous studies have used US for forensic examination of other skeletal regions in subjects from 10–30 years of age applying different classification systems [18, 43–59]. In our study, we used a newly designed three-stage system to rate the degree of epiphyseal closure of the knee. The US grading system is closely related to the MRI-based classification system of the knee used for age determination previously published by Jopp et al [35, 62]. As a modification to the MRI method, the US method only focuses on the superficial portion of the physis and disregards the unassessable central portion of the growth plate. This approach is common to US classifications of other anatomical regions and reflects the continuing ossification process regularly appearing at the central portion of the physis and gradually involving the more peripheral growth plate [18, 46, 48, 49, 54–59]. A refinement to previous staging systems in our study is the standardized recording of US volumes by translational motion along the physis. The resulting high–spatial resolution images allow a detailed assessment of the superficial physeal plate and can be independently performed offline by multiple readers.

Our study showed that our defined US stages are valid and have a high inter-rater agreement. The calculated US values correlate well with the MRI staging values. In particular, a completely open physis (stage 1) was staged with very high reliability. On the other hand, partially open and closed physis (stages 2 and 3) were more difficult to distinguish by both US and MRI. Upon comparison of the ability of the US and MRI methods to identify age groups, US staging showed a clearer separation of ages with less overlap. This could be due to the fact that, in the MRI method, assessment of stages was carried out only on the basis of a representative layer, whereas, in the US method, an estimate of an entire sequence along the physeal plate could be made.

In clinical use, the bone age is compared with the patients’ chronological age to define skeletal maturation. Yet, in forensic age estimation with unknown chronological age, signs of skeletal ossification are used to define an upper or lower age limit of an individual. In our study, US worked in particularly well for forensic staging of younger individuals. The US method can determine that a male individual is < 18 years of age if classified stage 1 (maximum age 16.5 years) and stage 2 (maximum age 17.5 years). Upon comparison to our MRI data and applying a stage 2 threshold, the sensitivity of the US method for definition of minority was higher (100% vs. 70%) with slightly lower specificity (70% vs. 85%). Interestingly, the mean and maximum ages of the MRI stages 1–3, which correspond well to previous MRI studies, were significantly higher than those of the corresponding US stages [28, 36, 37, 39, 64]. Accordingly, Fan et al demonstrated higher mean and maximum ages when staging was based on MRI instead on x-ray examinations [34]. These differences are explained by the better contrast and definition of MRI and its capacity detect even very small central growth plate remains. The higher detail and complexity of the ossification process as depicted by MRI may also pose problems to visual grading systems and explain the relatively lower inter-rater agreement for the femur in our study.

Following this line of argument, the ease to produce and to interpret high-resolution scan of the peripheral growth plate at the knee is a clear strength of the US method. The time required to examine the knee by US using the introduced method was relatively low (2.65 ± 0.91 min) and significantly shorter than examination by MRI (24.72 ± 2.72 min). The difference in the duration between the US and MRI methods should be even more pronounced because proper positioning of patients for MRI is time-consuming and was not measured in our study. Furthermore, the time spent for an US may be reduced further by limiting the exam to one scan per physis because we did not find any differences in staging when comparing recordings from both views. Other factors in favor of staging by US include that some patients are not eligible for MRI (in our study 4 cases, 10%) and MRI is more costly and less available than US.

Our study has the following limitations:

1. In this feasibility study, a relatively small homogenous sample with a restricted age range was examined. The results should be validated with a larger cohort and female subjects and persons with different ethnic and socioeconomic status should be included
2. Variations in epiphyseal growth patterns, such as epiphyseal cortical irregularities, and body composition, such as obesity, that can lead to misleading or less sensitive assessments by US alone need to be identified.
3. Cases of unknown skeletal pathology, underlying diseases or abnormalities, such as nutritional or hereditary growth plate disturbances, autoinflammatory disorders, overuse injuries, or trauma, may be missed by a focused US only assessment. In our study, strict exclusion criteria were applied; however, in a clinical situation and with a more heterogenous population under investigation, the US method must always be cautiously applied and may require the use of additional evaluation methods.
4. Ultrasound is very operator-dependent and therefore less objective compared with CT or MR. The introduced method can improve the accuracy of age determinations because it includes skeletal maturation criteria based on standardized US volumes.
  In summary, we can conclude that even though the time requirement is substantially lower for US than for MRI, US can reliably assign individuals to different ossification stages with clear chronological age separations. The US method can determine that a male individual is < 18 years of age if classified stage 1 or stage 2 (maximum age 17.5 years), and may therefore be used as a non-invasive imaging tool in forensic age estimation to identify younger individuals without using ionizing radiation. More studies with larger numbers of adolescents and young adults are needed to further evaluate this method for forensic estimation in view of legal proceedings. Thus far, the correlation between age and classification (18 years: yes/no) has been calculated based on the MRI and US visual staging systems. Potentially, future artificial intelligence (AI)–based classification systems can improve the level of detail in staging by not being restricted to only three stages.

## Electronic supplementary material

ESM 1 US volume stage 1 Right knee of a 15-year-old boy (same case as in Figure 2) showing an open growth plate on the longitudinally oriented 2D US volume of the lateral proximal tibia (US-S1). Note the hook-like configuration of the ossified cortex continuing into the physeal plate. (AVI 20398 kb)

ESM 2 US volume stage 2 Right knee of a 17-year-old boy (same case as in Figure 3) showing a slightly open growth plate on the longitudinally oriented 2D US volume of the lateral proximal tibia (US-S2). (AVI 47623 kb)

ESM 3 US volume stage 3 Right knee of a 17-year-old boy (same case as in Figure 4) showing a closed growth plate on the longitudinally oriented 2D US volume of the lateral proximal tibia (US-S3). (AVI 49584 kb)

## Abbreviations

2d: Two dimensional
CK: Cohen’s kappa
MRI: Magnet resonance tomography
MRI-S1: MRI stage 1
MRI-S2: MRI stage 2
MRI-S3: MRI stage 3
PoA: Percentage of agreement
US: Ultrasound
US-S1: US stage 1
US-S2: US stage 2
US-S3: US stage 3
